# Phantomless calibration of glenoid trabecular bone density: improved accuracy using age-stratified reference tissue densities

**DOI:** 10.1093/jbmrpl/ziag112

**Published:** 2026-07-14

**Authors:** Maximilian Modelhart, Dennis C Agbanyim, Davide Cester, Philipp Moroder, Stephen J Ferguson, Eva C Herbst

**Affiliations:** Paracelsus Medical University (PMU), 5020 Salzburg, Austria; Department of Shoulder and Elbow Surgery, Schulthess Klinik, 8008 Zurich, Switzerland; Department of Health Sciences and Technology, Institute for Biomechanics, ETH Zurich, 8006 Zurich, Switzerland; Institute of Diagnostic and Interventional Radiology, University Hospital Zurich, 8091 Zurich, Switzerland; Department of Shoulder and Elbow Surgery, Schulthess Klinik, 8008 Zurich, Switzerland; Department of Health Sciences and Technology, Institute for Biomechanics, ETH Zurich, 8006 Zurich, Switzerland; Department of Shoulder and Elbow Surgery, Schulthess Klinik, 8008 Zurich, Switzerland; Department of Health Sciences and Technology, Institute for Biomechanics, ETH Zurich, 8006 Zurich, Switzerland

**Keywords:** phantomless calibration, glenoid bone density, age stratification, fatty infiltration, quantitative CT, density variation in reference tissue

## Abstract

Evaluation of glenoid trabecular bone quality is important for patients undergoing shoulder arthroplasty, as the density of the glenoid bone is likely to influence implant stability. Routine CT scans show promise for quantitative bone density assessments, but CT-based calibration typically involves scanning a phantom, which limits its application in clinical practice and in retrospective research studies. Here, we developed and validated a phantomless BMD measurement technique to evaluate glenoid trabecular bone density (mg/cc) using clinical shoulder CT scans of 88 patients (48 males, 40 females; mean age: 53 years). Air, fat, and muscle tissue were used as internal density references to develop patient-specific calibration functions for glenoid BMD estimation. The phantomless calibration was validated by comparison with an asynchronous phantom-based calibration using the European Spine Phantom. A Bland-Altman analysis yielded a mean difference of 0.37 mg/cc (95% CI: [–1.39, 2.14] mg/cc) between the two methods. Further improvement in internal calibration accuracy was achieved by implementing an age-stratified approach (two age groups; cut-off 55 years) to account for age-related declines in muscle density. With age stratification of reference tissue densities, the mean difference between internal and phantom-based calibration was reduced to 0.20 mg/cc (95% CI: [–1.38, 1.78] mg/cc). The proposed phantomless calibration approach provides a robust and clinically feasible method to evaluate glenoid trabecular bone density in both research and clinical applications. Moreover, the results highlight the importance of considering patient-specific variations in shoulder muscle density to ensure accurate internal calibration.

## Introduction

The quality of the glenoid bone is critical for stable fixation of the glenoid implant component in shoulder arthroplasty. Among individuals, there exists a high variability in glenoid bone density, depending on age, sex, and glenoid morphology.^1–3^ This variability in bone density likely contributes to glenoid loosening, which is a common postoperative complication in anatomical total shoulder arthroplasty (aTSA).^2^

Computed tomography (CT) scans are routinely performed for preoperative evaluation and surgical planning in patients receiving shoulder arthroplasty. In addition to providing information on shoulder morphology, CT scans produce a three-dimensional density distribution image allocating a specific density value, in Hounsfield Units (HU), to each voxel. The relationship between HU and physical density is approximately linear.^3^ However, the HU scale must be understood as a scale of relative rather than absolute values, which is influenced by scan acquisition parameters, particularly tube voltage, reconstruction kernel, and scan manufacturer.^3^ This means that tissues of the same density may yield different HU values depending on the underlying scan parameters, making HU an unreliable and highly scan-specific density measure for comparison across patients or studies.^4^

Despite these limitations, several studies have used HU values as a proxy for BMD to investigate the relationship between glenoid bone quality and glenoid loosening, with contradictory findings.^5^ Although a correlation between glenoid bone quality and the mechanical responses of the implant-bone interface could be verified in a finite element analysis^5^ and lower glenoid bone density was associated with implant loosening in a cadaveric study,^6^ a retrospective clinical study could not show a clear relationship between glenoid bone density and aseptic implant loosening.^7^ However, the clinical study included CT scans from several scanners without uniform tube voltages and reconstruction kernels. As these variations directly affect HU values, the resulting inconsistencies may obscure the true influence of bone density on glenoid loosening. Therefore, further investigation of this relationship is required using a more robust and standardized evaluation approach.

In quantitative computed tomography (QCT), the HU values are converted into volumetric BMD (vBMD) values, which not only provides an absolute, standardized measure of density, but also reduces the effect of scan parameters through calibration.^8^^,^^9^ Calibration phantoms are considered the gold standard in QCT,^10^ as they serve as a physical reference (in hydroxyapatite or liquid dipotassium phosphate) for the conversion of HU into vBMD.^11^ In synchronous calibration, phantoms are scanned together with the patient, providing a scan-specific calibration function. However, clinical CT scans are not routinely performed with calibration phantoms due to additional costs and workflow limitations.^10^ In addition, synchronous scanning of a phantom increases the overall density of the scanned volume, which in turn increases the radiation dose needed. Even if the radiation increase is only minor, changing routine scan protocols for patients typically requires ethical approval. Asynchronous phantom calibration offers an alternative in which the phantom is scanned separately using the same scan protocol as in the patient scans, and the resulting calibration function is then applied to derive vBMD values from patient data. This is a promising approach to assess bone density without increasing patient radiation exposure, considering the high agreement between asynchronously and synchronously calibrated vBMD estimates.^12^^,^^13^ However, the generated calibration function is protocol-specific rather than patient or scan-specific^10^ and is only valid if scanner stability is ensured.^12^ This restricts its applicability in retrospective or multicentric studies, where scan acquisition parameters often vary and information on scan stability is frequently unavailable.

Given these constraints, internal (phantomless) calibration methods have been proposed to convert HU values into vBMD values based on internal reference tissue densities rather than a physical phantom.^14^ HU values of reference tissues (eg, air, muscle, subcutaneous fat, and aortic blood) are extracted and plotted against their corresponding densities (in hydroxyapatite/BMD equivalence) to derive a linear regression function, which is then used to calibrate bone mineral density in the region of interest.^15^^–^^17^ Previous studies have validated internal calibration methods for the femur, hip and spine regions, confirming high agreement with phantom-based vBMD calibration.^15^^,^^18^ Internal calibration combines the advantages of both synchronous and asynchronous phantom-based calibration by providing a scan-specific calibration without additional radiation exposure, costs, and workflow limitations. Additionally, it can be applied to retrospective datasets, overcoming a key limitation of phantom-based calibration.

Phantomless calibration has only recently been implemented in the shoulder region.^19^ To evaluate glenoid vBMD, Ritter et al.^20^ used reference tissue densities derived from the femoral region^15^ rather than shoulder-specific values. However, rotator cuff muscles are strongly affected by age-related fatty infiltration, a well-described process that alters muscle tissue density. This could compromise internal calibration accuracy if not taken into account.^17^

To our knowledge, no study has validated an internal calibration for glenoid vBMD using shoulder-specific reference tissue densities. Therefore, the objectives of our study were: (1) to establish and validate a shoulder-specific phantomless calibration method for glenoid trabecular vBMD, and (2) to implement an age-stratified internal calibration approach to account for potential age-related variation in reference tissue density, particularly in muscle tissue. We hypothesized that phantomless glenoid trabecular vBMD calibration shows high agreement with phantom-based calibration. Furthermore, we expected an improved internal calibration accuracy when accounting for age-related density changes of reference tissues.

## Materials and methods

### Scan data

Although the aim of this study was to generate a method that can be applied to subjects receiving shoulder arthroplasty, this study was conducted using emergency subjects. The choice of an emergency room (ER) scanner enabled the collection of sufficient data using a consistent scan protocol for validation of the phantomless calibration method. This study was performed with ethics approval by the local ethics committee of Zurich (BASEC Nr.: 2015-00233). Written consent was obtained from all study participants.

#### Phantom CT scans

The European Spine Phantom (ESP; QRM, Germany) was used for asynchronous vBMD calibration to validate the phantomless method. Asynchronous phantom calibration using the ESP yields good accuracy and precision for quantifying trabecular vBMD in the spine, when compared to synchronous phantom calibration.^13^ Furthermore, synchronized phantom scans were not logistically feasible in emergency CT scans and even in the clinic would have required additional ethical approval.

The ESP is a semi-anthropomorphic phantom that contains a spine insert consisting of three manufactured vertebrae surrounded by a resin-based, water-equivalent phantom torso.^21^ The trabecular bone regions of vertebrae 1–3 from the ESP phantom were used for the phantom-based calibration; these regions represent different trabecular densities (HA values of 50, 100, and 200 mg/cc). The different densities of the components are standardized and achieved by adding specific amounts of calcium hydroxyapatite (HA) to a water-equivalent mixture in the manufacturing process. The manufacturer reports an accuracy of +/– 5% for the specified calcium HA values.

Between August 2023 and October 2024, the ESP was scanned a total of 20 times with the trauma room CT scanner (SIEMENS SOMATOM Force, Siemens Healthineers International AG, Germany) in the emergency unit of Zurich University Hospital using the hospital’s standard shoulder scan protocol. Initially, the phantom was scanned after each patient and at least once a week. After confirming the stability of the CT scanner, weekly phantom scans were carried out to ensure long-term stability and eliminate the possibility of drift. Stability was assessed by checking the HU variability of the three trabecular bone segments in the phantom scans over time. The last phantom scan was conducted 11 mo after the second to last scan. All phantom scans were acquired in the axial plane at 120 kVp, with a Br59s reconstruction kernel and a slice thickness of 1 mm. Pixel spacing ranged from 0.174*× *0.174 to 0.365*× *0.365 mm across the 20 scans.

#### Patient CT scans

Of the 121 ER patients who underwent a shoulder CT scan at Zurich University Hospital between August 2023 and October 2024, 88 patient scans were included in the analysis. 24 scans were excluded due to the presence of a shoulder implant, bone screws and/or plates in or near the scapula, or complex fractures of the glenoid or proximal humerus. Two patients were excluded due to advanced osteoarthritic degeneration of the glenohumeral joint, which precluded segmentation of the glenoid region of interest. Four patients were excluded because of either low scan quality or because the acquired CT scans exhibited spatial misorientation, preventing accurate segmentation of all tissues of interest. Three patients were excluded due to CT scans acquired at tube voltages that differed from the standard protocol. CT-scans of all patients included in the final study sample were performed on the same CT scanner as the phantom scans using the same imaging protocol at 120 kVp and a Br59s reconstruction kernel. The slice thickness of the acquired CT scans ranged from 1 to 2 mm and the pixel spacing ranged from 0.162*× *0.162 to 1.129*× *1.129 mm. The age range of the patients was 20-85 years (mean age 53.2 years, SD 18.4 years). The patient cohort consisted of 48 male and 40 female patients.

### Segmentation

All segmentations were performed in 3D Slicer.^22^ To segment the trabecular regions of the phantom, a single phantom scan was first manually segmented, with each trabecular region as a separate segment. Then, to ensure consistent segmentation of trabecular regions between scans, each new target scan was thresholded to create a model of the entire phantom. The template including trabecular segment masks was then transformed to the target phantom segmentation using iterative closest point (ICP) registration with VTK in Python (3.13.0). This enabled consistent placement of trabecular segments between all phantoms.

**Figure 1.**
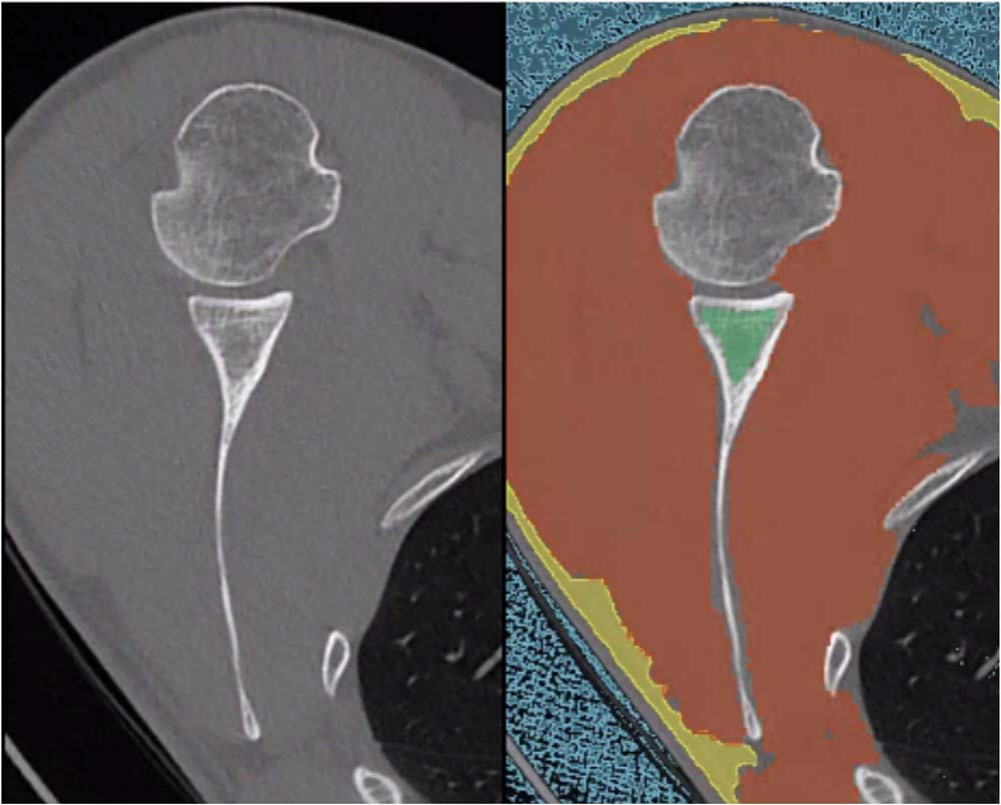
Axial CT slice of a right shoulder. Original gray-scale image on the left, with the corresponding referene tissue segmentations on the right, demonstrating air (blue), fat (yellow), muscle (red) used for density calibration of the glenoid trabecular bone (green). The air region of interest (RoI) was semi-automatically segmented based on defined thresholds (–1023 HU to –970 HU; –1024 (lowest detectable HU number) was spared to prevent inclusion of empty image regions). The muscle ROI was automatically segmented using TotalSegmentator, while the subcutaneous fat and glenoid ROIs were automatically segmented using internally trained deep-learning models. This single axial slice is shown for visual purposes; all reference tissues were segmented in the entire CT scan, where anatomically present.

For each patient scan, the segmentation of air, subcutaneous fat, skeletal muscle, and glenoid trabecular region of interest was performed (Figure 1). The air region of interest was semi-automatically segmented using a Python script applying a threshold. The lower and upper borders of the threshold range (–1023 HU to –970 HU) were selected to include air-specific density voxels (per definition –1000 HU) and to account for possible variability in the scanning process and image reconstruction. The lowest CT number used by the scanner (–1024 HU) was excluded to prevent segmentation of empty image regions in the field of view, which display artificial CT values but no physical density. The upper threshold was defined conservatively to prevent including non-air tissue. This approach allows for incomplete segmentation of the entire air tissue in the scan, but ensures that all included voxel represent air-specific density. Skeletal shoulder muscles were automatically segmented using TotalSegmentator,^23^ implemented in 3D Slicer.^22^ Subcutaneous fat and glenoid ROI were segmented using two internally trained deep-learning models within the MonaiLabel^24^ extension in 3D Slicer, based on SegResNet architecture. For model training, manually segmented subcutaneous fat and glenoid ROI from 30 randomly selected patients from the 2023 scans were used. Each model was trained for 1000 epochs. The trained models were then applied to segment the subcutaneous fat and glenoid ROI in the remaining patient scans. All model-generated segmentations were manually reviewed by an orthopedic resident (M.M.) to ensure correctness. In total, three subcutaneous fat and four glenoid segmentations required manual editing. In 9 patients, automated segmentation either did not produce any output or resulted in severely deficient segmentations of the glenoid ROI, the subcutaneous fat, or both regions. In these cases, segmentation was performed manually. Since the goal of the automated segmentation models was to speed up processing, reduce bias, and test the feasibility of complete automation, these manual adjustments were deemed acceptable as they do not affect the validation of the phantomless calibration.

### Peak detection

**Figure A1.**
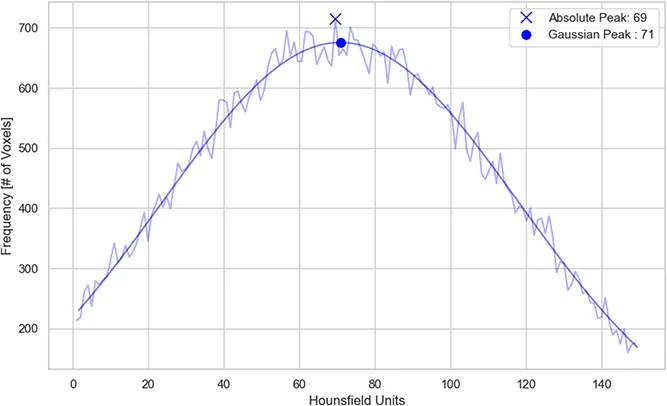
Example histogram of peak detection demonstrating the difference between absolute and gaussian peaks.

To extract the HU values of the segmentations, an adaptation of the histogram and the peak fitting approach (see Eggermont et al.^15^) was used in this study. A histogram of the HU voxel intensities was created for each segment of each phantom and patient scan over a predefined HU range expected to include the characteristic HU values of the respective tissues (air: –1023 to –970 HU; fat: –250 to 100 HU; muscle: –100 to 200 HU; glenoid trabecular bone: –250 to 600 HU). A Gaussian fit was then plotted using the curve_fit function of the SciPy package in Python (version 3.13.0) to obtain the maximum HU (Gaussian Peak) for each segment of all phantom and patient scans. This approach is more robust against slight segmentation errors than using the mean and better represents the spread of the data in the peak region than using the absolute peak (Figure A1).

### Phantom-based calibration functions

**Figure A2.**
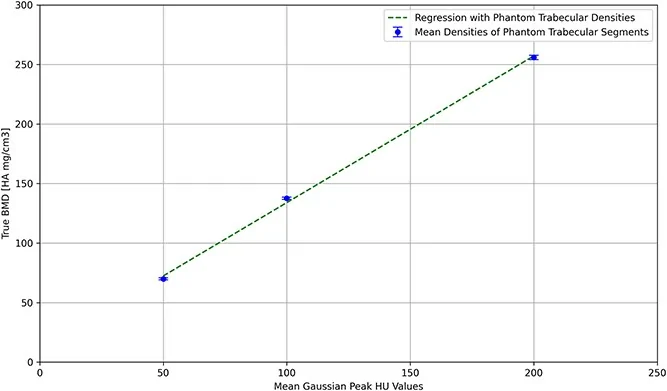
Linear regression function for phantom-based BMD calibration.

To construct the asynchronous phantom calibration function, the mean Gaussian Peak HU value of each trabecular vertebrae segment was extracted from all phantom scans. These values were then plotted against their corresponding HA content-based BMD values, as reported by the manufacturer, to derive a linear regression (see Figure A2).

### Phantomless calibration functions

To establish the reference tissue densities (air, fat, and muscle), we used the phantom-based calibration equation to convert the tissue’s HU values into vBMD equivalence (in HA mg/cc). Although these tissues do not contain hydroxyapatite, we use this equivalence to represent overall density values to generate the calibration equations, from which the vBMD of the bone can be extrapolated. We used a leave-one-out approach so that for each patient, the density values of the reference tissues included the mean tissue density values of all other scans except that particular patient. In this manner, we can test how the method would perform on a new patient whose tissues were not part of the reference tissue density calibration dataset. Additionally, this maximizes the dataset for density calibration of reference tissues, reducing the susceptibility to outliers and selection bias that would arise from dividing patients into training (reference tissue) and testing (bone calibration) datasets. To construct a patient-specific phantomless vBMD calibration function, a linear regression was fitted for each scan between the peak HU values of the patient’s air, subcutaneous fat and skeletal muscle segmentation, and the calculated HA equivalence for air, subcutaneous fat, and skeletal muscle tissue from all other subjects.

The effect of age on reference tissue densities was empirically tested within our sample, confirming whether subcutaneous fat and muscle tissue densities show age-related changes. To account for age-related density variation, we developed a second phantomless calibration method, using age-stratified reference tissue densities (ie, for each patient, the reference tissue densities were derived from patients in the same age group, rather than the whole sample). We defined 55 years as the cut-off value to separate between younger and older subjects. This threshold was chosen because structural changes in the rotator cuff muscles, such as fatty infiltration and muscle atrophy, become more pronounced from midlife (45–60 years) onward.^25^ Considering the age distribution within our sample and the mean patient age, this cut-off yielded two groups of comparable size (43 vs 45 patients).

### Statistical analysis

Descriptive statistics were used to evaluate CT-scan stability by calculating the mean Gaussian peak HU values for trabecular vertebrae segments 1-3 across all 20 phantom scans, together with standard deviation (SD) and range. Additionally, the coefficient of variation (CV) was calculated to provide a relative measure of variability. Tissue peak HU values and phantom-based HA values of all tissues were summarized by reporting mean, SD, and range. The relationship between patient age and phantom-calibrated HA density values for muscle, fat, and glenoid ROI was assessed using Pearson correlation and regression analysis. Differences between age groups were assessed after testing for normality (Shapiro-Wilk test) and homogeneity of variance (Levene test). Group comparisons were performed using Student’s *t*-test (normally distributed data with equal variance), Welch’s *t*-test (normally distributed data with unequal variance), or Mann-Whitney U test (non-normally distributed data). The agreement between phantom-based and phantomless vBMD calibration was assessed using Bland-Altmann analysis. Bland-Altman analysis quantifies the mean difference and limits of agreement, representing the interval including 95% of the differences between two measurements.^26^ The normality of pairwise differences was verified using Q–Q plots and the Kolmogorov–Smirnov test. The intraclass correlation coefficient (ICC3, two-way single fixed raters, based on Shrout and Fleiss 1979) was also calculated as an additional metric of agreement. All statistical analyses were performed using Numpy, SciPy, Matplotlib, Pingouin packages in Python (version 3.13.0). A *p*-value <.05 was considered statistically significant. All Python scripts used in this study for image analysis and statistics were created by the authors for internal use and are not publicly available.

## Results

### CT-scanner stability

The coefficient of variation for the Gaussian Peak (HU) values of all vertebrae segments was below 1.2% across all phantom scans and absolute standard deviations were low (0.83–1.80 HU). This confirms high CT scan stability (see Table 1 and Figure A8).

**Table 1:** Evaluation of CT-scanner stability by comparing HU values across phantom scans.

Segment name	Gaussian peak (HU)	Std (HU)	Min (HU)	Max (HU)	Coefficient of variation in %
**Trabecular vertebra 1**	69.95	0.83	68	71	1.18
**Trabecular vertebra 2**	137.6	0.94	136	140	0.68
**Trabecular vertebra 3**	255.90	1.80	253	260	0.70

### Inter-patient reference tissue density variation

#### HU values

The air samples showed the lowest density variation across patients, with a SD of 3 HU and a range of 15 HU. Note that the standard variation in air peaks serves as a baseline to account for any scan-specific variation regardless of patient anatomical variation. Skeletal muscle tissue showed a standard deviation of 7 HU and a range of 33 HU, while subcutaneous fat showed a standard deviation of 15 HU with a range of 92 HU. The glenoid trabecular bone exhibited a SD of 80 HU and a range of 351 HU (Table 2 ).

**Table 2:** Tissue specific density values in Hounsfield units (HU).

	Glenoid bone peak	Air peak	Skeletal muscle peak	Subcutaneous fat peak
**Gaussian peak (HU)**	197.48	–992.34	46.02	–93.65
**Std (HU)**	79.57	2.93	6.98	15.22
**Lowest value (HU)**	35	–999	25	–113
**Highest value (HU)**	386	–984	58	–21

### Phantom calibrated density in HA equivalence

Phantom-calibrated vBMD equivalence corresponding to the respective HU values are summarized in Table 3. The results of age-related differences in muscle, fat, and glenoid HA equivalence are summarized in Table 4.

**Table 3:** Phantom-calibrated density values of all tissues in hydroxyapatite (HA) equivalence (mg/ cc).

	Glenoid bone peak	Air peak	Skeletal muscle peak	Subcutaneous fat peak
**Mean (HA)**	151.54	–813.44	28.70	–84.57
**Std (HA)**	64.53	2.38	5.66	12.34
**Lowest value (HA)**	19.76	–818.84	11.65	–100.27
**Highest value (HA)**	304.43	–806.67	38.42	–25.65

**Table 4:** Comparison of phantom-calibrated densities (mg/cc) between age groups.

	<55 years (*n* = 43)	>55 years (*n* = 45)	*p*-value
**Muscle (HA)**	32.48 (3.77 SD)	25.10 (4.74 SD)	<.001
**Fat (HA)**	–82.43 (12.56 SD)	–86.63 (11.91 SD)	.039
**Glenoid bone (HA)**	198.45 (54.33 SD)	106.70 (34.78 SD)	<.001

**Figure A3.**
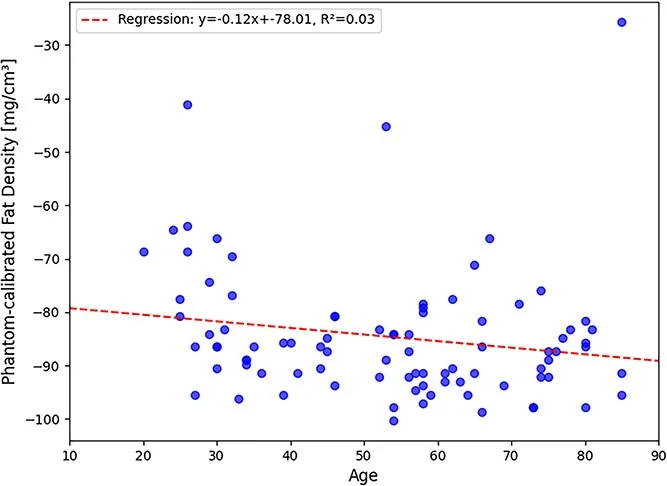
Scatterplot with linear regression: phantom-calibrated fat density vs age.

**Figure A4.**
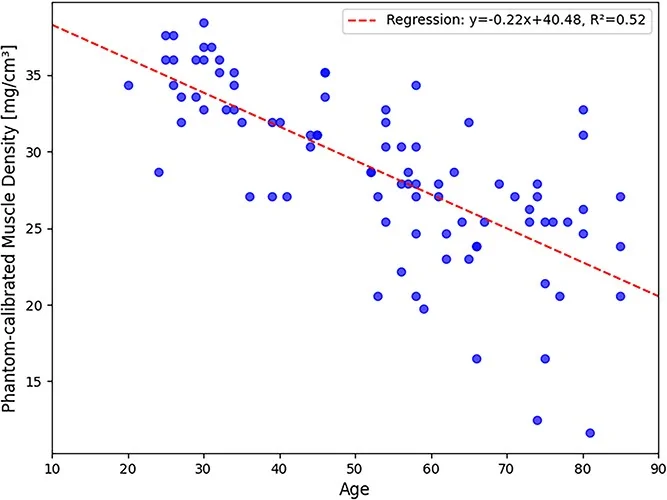
Scatterplot with linear regression: phantom-calibrated muscle density vs age.

Muscle density showed a strong negative correlation with age (*r* = –0.718, *p* <.001), while fat density did not show a significant correlation with age (*r* = –0.183, *p* =0̇.087) (see Figures A4 and A3). However, when comparing between the age groups, muscle and fat densities were significantly higher in patients younger than 55 years (*p* <.001 and *p* =0̇.039, respectively; see Table 4), although the effect in muscle was more pronounced.

**Figure A5.**
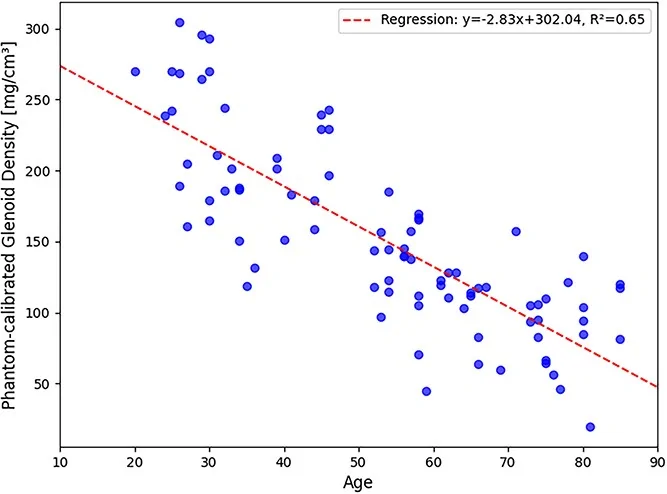
Scatterplot with linear regression: phantom-calibrated glenoid BMD vs age.

**Figure A8.**
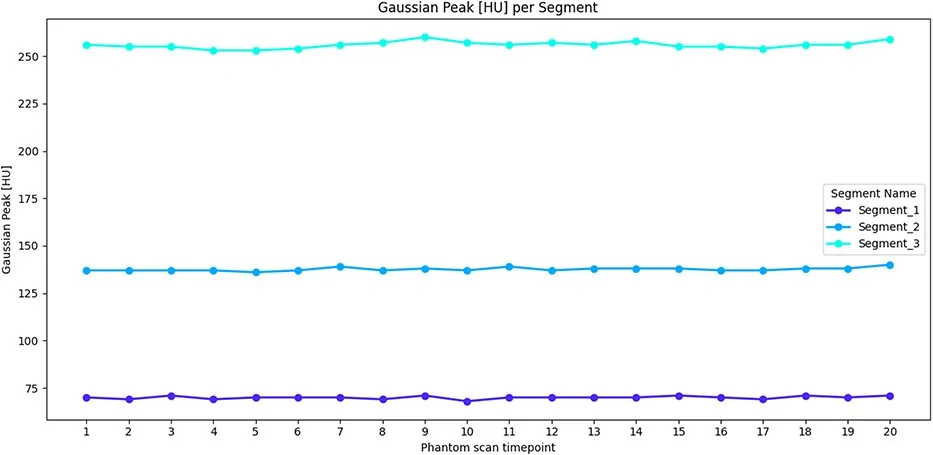
Multiple-line plot of trabecular vertebra density distribution showing low variation between repeated phantom scans.

Phantom-calibrated glenoid vBMD decreased with patient age (*r* = –0.804, *p* <.001), as shown in Figure A5. Patients younger than 55 years of age had significantly higher glenoid vBMD than patients older than 55 years (*p* <.001), see Table 4.

### Phantom-based versus phantomless glenoid vBMD calibration

**Figure 2.**
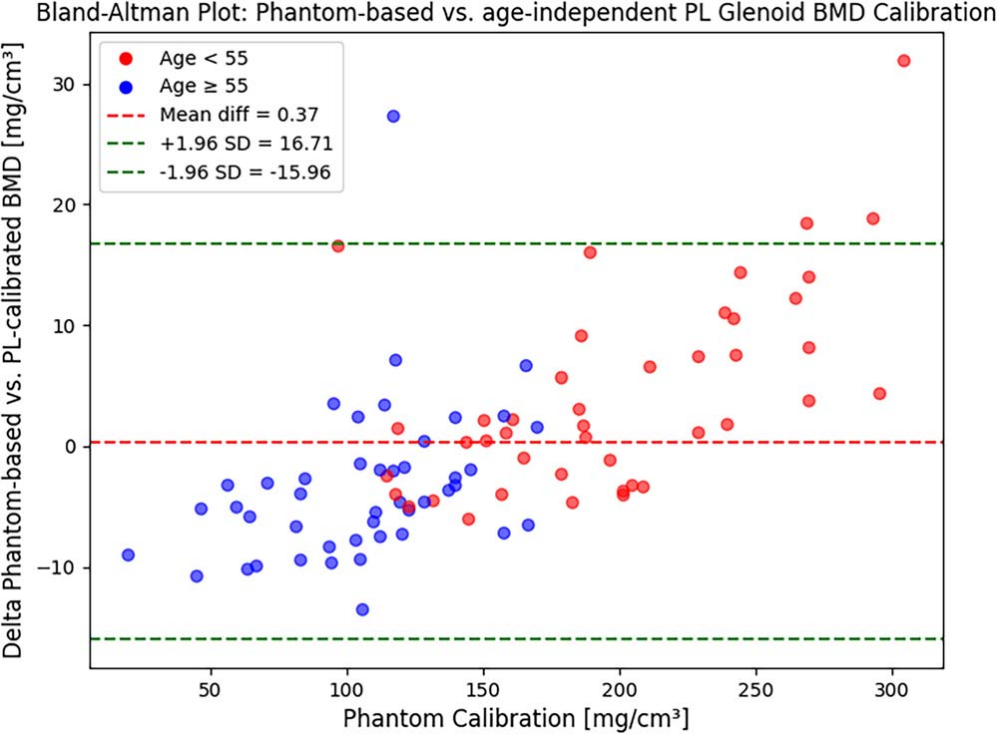
Bland-Altmann analysis: difference between phantom-based and age-independent phantomless (PL) glenoid vBMD calibration.

Without age-stratification, the ICC between both calibration methods was 0.991 (95% CI: [0.986, 0.994]). The mean difference was 0.37 mg/cc (95% CI: [–1.39, 2.14] mg/cc), and the limits of agreement ranged from –15.96 mg/cc (95% CI: [–18.95, –12.98] mg/cc) to 16.71 mg/cc (95% CI: [13.72, 19.69] mg/cc). The Bland-Altman analysis revealed a positive proportional bias between phantom-based and non age-stratified phantomless calibrated glenoid vBMD estimates. With increasing glenoid vBMD, the phantomless method tended to underestimate density values when compared to the phantom-based calibration, whereas the opposite trend was observed at lower bone densities. Consequently, given that younger patients tended to have higher densities, the underestimation of glenoid vBMD predominantly affected younger patients, whereas the overestimation predominantly affected older patients (see Figure 2).

**Figure 3.**
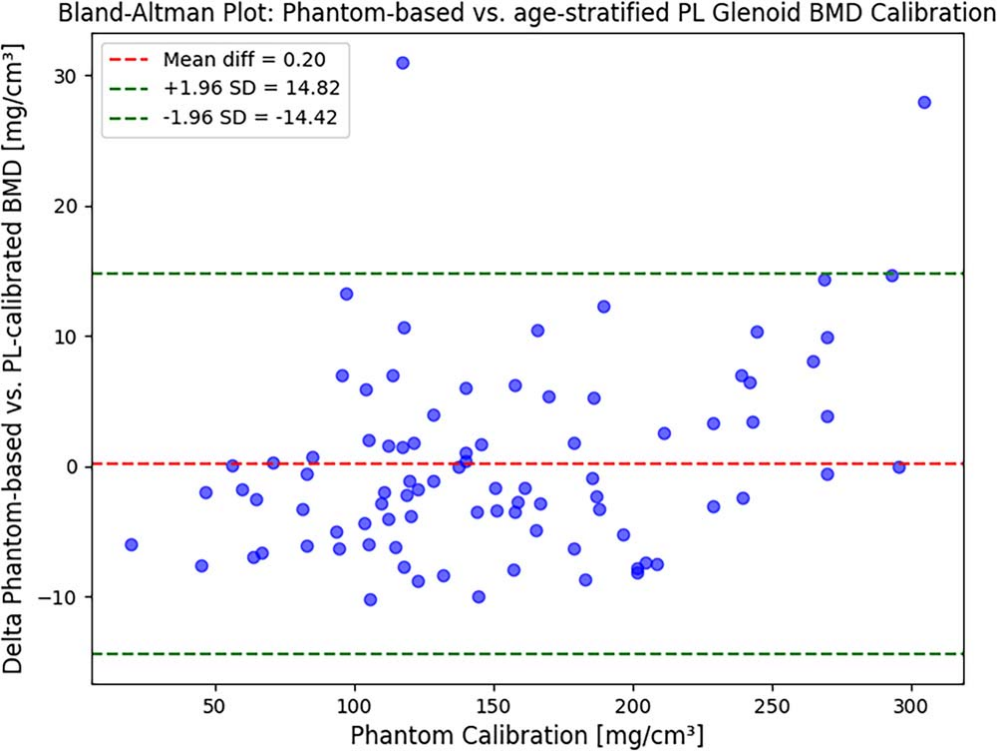
Bland-Altmann analysis: difference between phantom-based and age-stratified phantomless (PL) glenoid vBMD calibration.

With age-stratification, the reported ICC between both calibration methods was 0.993 (95% CI: [0.989, 0.995]). The mean difference was 0.20 mg/cc (95% CI: [–1.38, 1.78] mg/cc) and the limits of agreement ranged from –14.42 mg/cc (95% CI: [–17.09, –11.75] mg/cc) to 14.82 mg/cc (95% CI: [12.14, 17.49] mg/cc). The Bland-Altman analysis revealed that, after age-stratification, the proportional bias between the phantom-based and phantomless calibration was substantially reduced, as shown in Figure 3.

## Discussion

The main findings of our study are that (1) the phantom-based and phantomless calibrated vBMD estimates of the glenoid trabecular bone yield similar results, and (2) an age-stratified approach further improves the accuracy of phantomless calibration by accounting for age-related changes in muscle tissue density.

Our results indicate excellent reliability and high agreement between the phantomless calibration and the asynchronous, phantom-based calibration of glenoid trabecular bone density. However, a proportionality effect was observed in the age-independent model: phantom-based calibration yielded higher BMD values compared to the phantomless method for patients with higher glenoid bone densities, whereas the opposite trend was observed in patients with lower glenoid bone densities. This can be explained by the age-dependent variation in reference tissue densities, particularly muscle tissue, as shown in Table 4 and Figure A4. Since, in the patient-specific calibration function, the reference BMD equivalent for muscle tissue was derived from the phantom-based density values of all other patients in the sample, any deviation of a subject from the mean reference tissue densities could lead to over- or underestimation of the individual’s muscle density, and consequently, glenoid vBMD. Because of the age-related decline in muscle density, most likely due to fatty infiltration, the age-stratified phantomless calibration was able to reduce the proportional bias and increase the reliability and measurement agreement with the phantom-based method. The limits of agreement ranged from –14.42 – 14.82 mg/cc, corresponding to a relative deviation of +/– 10% compared with the mean trabecular glenoid vBMD of roughly 150 mg/cc. Future studies are needed to investigate mechanical and clinical consequences of this measurement deviation in vBMD. Overall, the narrow limits of agreement indicate a high measurement accuracy for the age-stratified phantomless calibration method across a clinical spectrum of glenoid trabecular bone densities.

Variability in reference tissue density significantly influences phantomless calibration accuracy, as shown by Bartenschlager et al.,^17^ who discouraged the use of skeletal muscle tissue as an internal reference in elderly patients due to varying degrees of fatty infiltration. Our approach addresses this problem by using two different reference tissue populations to consider age-related density changes. Although we also observed significantly different subcutaneous fat densities between age groups, age-stratification of fat density appears less decisive for the increased accuracy of the age-stratified phantomless calibration approach, as the correlation between fat density and patient age was only weak and without statistical significance. Additionally, the relative impact of the density differences observed between age groups was greater for muscle than for fat. A potential explanation for the variability in fat density could be explained by varying amounts of body fat among patients. Higher BMI has been linked with lower CT values of subcutaneous fat, likely due to changes in adipose tissue composition.^27^^,^^28^ The density range of subcutaneous fat in our cohort is comparable to values in the literature, with a commonly reported density range for adipose tissue of –190 to –30 HU.^29^^,^^30^

Accurate segmentation of reference tissues is important for reliable HU extraction and, consequently, for phantomless calibration accuracy. In this study, we used an automated segmentation approach to segment the entire skeletal muscle and all subcutaneous fat visible in the scan using TotalSegmentator and a custom MonaiLabel segmentation model,^24^ respectively. In contrast, other studies have segmented small, localized volumes of interest (VOI), using rods, cylinders or polygons as segmentation masks to extract tissue-specific HU values.^14^^,^^17^^,^^31^ Using the entire reference tissue available has the benefit of using a substantially larger number of voxels for tissue-specific HU-value extraction, which may be more robust to regional density changes and small segmentation errors, compared to using a small VOI. This is particularly relevant in the shoulder region, where subcutaneous fat is a rather thin layer when compared to other anatomical regions such as the hip or the abdomen. The upside of smaller reference tissue VOI is that, if located close to the bone region of interest, they experience more similar beam hardening to the bone region of interest than tissues farther away from the bone. However, slight differences in small VOI positioning between scans may also cause variation in reference tissue HU since beam hardening artifacts vary within a scan.^17^ Automated glenoid ROI selection also decreases potential sources of bias when comparing densities across subjects. This is particularly relevant in longitudinal vBMD assessments, where highly reproducible segmentations are essential to evaluate tissue-specific density changes over time.^17^

The proposed shoulder-specific internal calibration method enables precise and fast assessment of glenoid trabecular vBMD and therefore represents a promising approach for both retrospective and prospective studies. The rationale of vBMD calibration is to minimize the impact of scan parameters and provide an absolute quantitative density measure for comparison across scanners and protocols.^10^

### Limitations

This study has several limitations. First, our sample consisted solely of trauma patients, who may introduce trauma-related tissue changes affecting density across all tissues. Although all patients with severe injuries to the proximal humerus/ glenoid region were excluded, minor post-traumatic changes (eg, subcutaneous haematoma) could theoretically alter tissue-specific densities. Automated muscle segmentation with TotalSegmentator might include small amounts of adjacent connective tissue and subcutaneous fat. However, as the segmentation approach was consistent for all patients across our sample, this is unlikely to explain the age-related changes in muscle density. The main reason for age-dependent decline in muscle density is more likely attributable to other factors, such as fatty infiltration.

Although converting HU to vBMD reduces the effect of tube voltage on density measures,^8^^,^^9^^,^^17^, sensitivity remains since vBMD estimates can be significantly influenced by reconstruction kernels.^14^^,^^32^ For context of the magnitude of such effects in the shoulder, a recent study using synchronous calibration of the acromion found average vBMD differences in standard and bone kernels of 3 mg/cc, although this effect did not reach statistical significance^33^. Notably, varying kernels have different impacts on cortical and trabecular bone densities. Giambini et al.^8^ found that sharpening kernels increase cortical HU and vBMD while decreasing trabecular HU and vBMD. The authors attribute their findings to an over- and under-shoot phenomenon due to enhanced spatial resolution in cortical bone, leading to an increased visualization of the bright cortex, whereas adjacent trabecular bone loses effective spatial resolution, leading to lower measured density values.^8^ Reconstruction kernels may likewise alter reference tissue densities and the derived calibration function. Thus, when using phantomless calibration in retrospective or longitudinal research, kernel-related variations must be considered to avoid confounding effects on vBMD evaluation.^14^ To account for this, several authors have promoted the use of standardized scan and reconstruction protocols.^9^^,^^32^^,^^34^ In this study, a uniform scan protocol was used, ensuring that any observed differences between the phantom-based and phantomless calibration are not attributable to external scan factors. Future studies should specifically investigate the effect of different reconstruction kernels on glenoid trabecular bone density and evaluate the consistency of internal calibration across various shoulder-specific scan protocols. While this study used the default emergency room CT-scan protocol for shoulder examinations as baseline for the comparison, the clinical introduction of our calibration method would require a more extensive assessment of the impact of the reconstruction parameters such as kernel choice, iterative reconstruction and metal artifact reduction (MAR) algorithms; particularly in the case of MAR, representative cases should be further investigated, as metal artifacts can significantly affect the HU accuracy and other relevant aspects of image quality.^35^^,^^36^

The ESP phantom carries inherent drawbacks. Constructed from a water-equivalent epoxy resin with added specific amounts of HA,^21^ it deviates from the actual bone anatomy. The water-equivalent epoxy resin used to suspend the HA in the phantom lacks accuracy in representing bone marrow, as real bone marrow contains not only water-equivalent components but also significant amounts of fat, which has a lower density than water. Furthermore, the ESP phantom is designed to calibrate abdominal CT scans and is not specifically configured for shoulder investigations. Variations in the phantom’s positioning compared to the isocenter as well as different amount and types of surrounding tissues, particularly other bones which would contribute to beam hardening, could in principle alter the measured HU values in the VOIs and affect the calibration. More realistic shoulder phantoms may actually increase agreement between our phantomless and phantom-based calibration, since the the attenuation and beam hardening patterns would more closely resemble those experienced by the tissues in the internal calibration. Similarly, drawbacks of the phantom such as the presence of an air gap may have contributed to the small variation seen between our phantom and phantomless calibration, and resolving such differences would improve agreement even further (in other words, while phantom-based validation is the gold standard, the phantomless approach resolves some limitations of the phantom-based approach).

It is important to note that any image-based calibration (HU, synchronous phantom-based, asynchronous phantom-based, and phantomless) will have a certain amount of error compared to ground truth (ash density) bone mineral density. One cause of this error is the presence of marrow fat between the trabecular bone. If an imaging voxel contains both marrow fat and trabecular bone, these two will be averaged by their relative contributions to the voxel (partial volume effect). Because of the low density of the marrow fat, this will underestimate the density of the bone within the actual trabeculae. However, the summed density of that voxel can still reveal an overall trend regarding stiffness of the region, which is important for simulating bone behaviour i.e. in finite element modeling. In other words, if more marrow fat is present, the apparent density and therefore the calculated stiffness of that voxel (and corresponding nodes/elements in the FE mesh) will decrease. Micro CT scans reduce partial volume effects through smaller voxel sizes (higher resolution), and enable assessment of bony microstructure at the trabecular level.^37^ However, such scans could not capture the whole shoulder in vivo and are not readily available in clinical practice. All current methods that are compatible with clinical routine CT scans (HU-based, synchronous and asynchronous phantom calibration, internal phantomless calibration) have partial volume effect issues. Although these limitations must be kept in mind, the aim of our method was to improve glenoid density estimation from routine clinical CT scans, which may facilitate both research and clinical decision-making in shoulder surgery. For example, patient specific evaluation of glenoid trabecular bone density could support pre-operative planning to optimize implant placement.

Our results highlight that the accuracy of phantomless calibration improves when incorporating patient age into the reference tissue density calibration. According to the ethical boundaries, our work was designed as a retrospective, fully anonymized study, and therefore most characteristics of the study participants are excluded by design from the analysis, with the exception of age and sex. Consequently, other potential clinical and demographic confounders, such as comorbidities or other patient-specific characteristics that affect the composition of the reference tissue, could not be assessed in this analysis. In addition, sex-specific subgroup analyses were not performed as further stratification of the cohort would have likely resulted in subgroup sizes too small to derive clinically meaningful density values. However, internal calibration accuracy could be further improved by incorporating age- and sex-specific reference tissue densities. Future studies with larger cohorts are needed not only to validate our proposed method but also to test its generalizability and the influence of additional patient-specific factors on internal calibration performance.

## Conclusion

This study demonstrates that a shoulder-specific internal vBMD calibration enables highly reliable and accurate evaluation of glenoid trabecular bone density in routine clinical CT scans. Furthermore, the calibration accuracy improved when accounting for age-related density changes in reference tissues, particularly muscle tissue, through the implementation of an age-stratified calibration approach. The proposed internal calibration method can be applied to both retrospective and prospective datasets, facilitating the assessment of glenoid trabecular bone density in various patient populations.

## Figures and Tables

**Figure A6.**
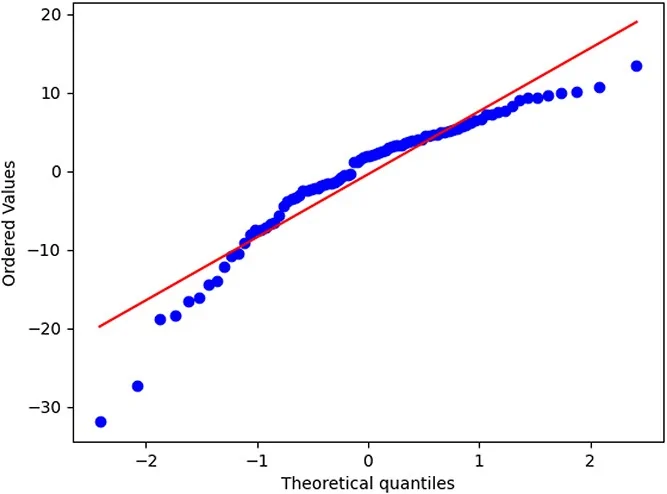
QQ-plot for differences in glenoid BMD between PL (age-independent) and phantom calibrated function.

**Figure A7.**
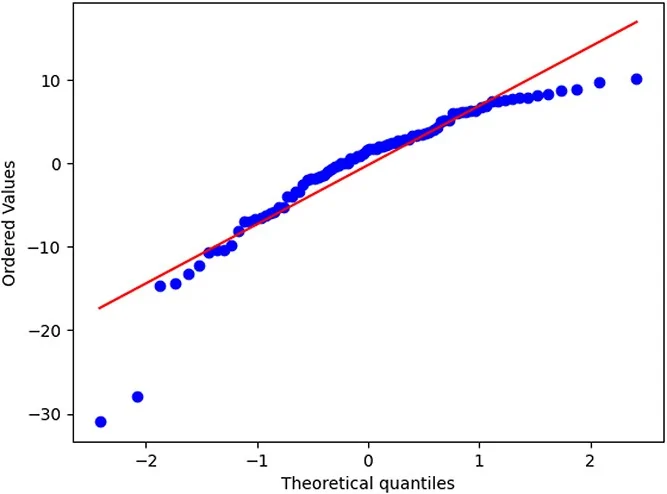
QQ-plot for differences in glenoid BMD between PL (age-stratified) and phantom calibrated function.

## Data Availability

Data are available upon reasonable request to the authors.
